# Some Reflections on Designing Effective Social Telerehabilitation Services for Older Adults

**DOI:** 10.5195/ijt.2016.6195

**Published:** 2016-12-15

**Authors:** GILBERTO MARZANO, VELTA LUBKINA, GATIS STAFECKIS

**Affiliations:** REZEKNE ACADEMY OF TECHNOLOGIES, REZEKNE, LATVIA

**Keywords:** Experiential learning, Healthy ageing, Social telerehabilitation services, Technologies and older adults, Usability of computer applications

## Abstract

The share of the population aged 65 years and over is increasing in every EU Member State, candidate country, and EFTA Member State. WHO/Europe has established a Subnetwork on Healthy Ageing within the European Healthy Cities Network and provides guidance to Member States in developing profiles of healthy ageing, since, given the increasing number of older adults, maintaining their health status is a crucial issue. This article, which is part of the Latvian national scientific program VPP INOSOCTEREHI, focuses on the issues related to the design, implementation, and assessment of user interfaces that can maximize usability in social telerehabilitation systems for older adult users. This is a very real challenge, since older adults don’t form a homogeneous class. Investigating older adults’ skills and their attitudes towards the perceived usefulness of computer applications could be helpful in identifying some sub-sets of older adult users and their typical behaviours.

Healthy ageing has been conceptualised from a number of different perspectives, both biomedical and social. It is more than just a matter of extension of life, and emphasizes quality of life as a key concern for health and social care services (Peel, Bartlett & McClure, 2004; Bartlett & Peel, 2005). It has also been observed that, in healthy ageing, the subjective dimension is particularly important: for an individual, it encompasses well-being, capacity for independent activity, meaningful involvement, supportive environments, and positive attitudes (Cutchin, 2005; Bowling, 2006; Stenner, McFarquhar & Bowling, 2011).

All this extends the sphere of the potential services for older adults and, in accordance with the diffusion of new technologies, enhances the opportunity to create and deliver new services based on Information and Communication Technology (ICT). ICT is an extended term for information technology and includes software and computer based-systems as well as internet applications which enable users to access, store, transmit, and manipulate information (Jones & Groom, 2011). Technology can reduce the decline of functions in older adults and support their rehabilitation activity, helping them to cope with daily life in a way more similar to their earlier (i.e., younger) lifestyles.

Social telerehabilitation (or social e-rehabilitation) is defined as the delivery of social rehabilitation services through telecommunication networks and the internet (Marzano, Lubkina & Rizakova, 2015).^1^ Figure 1 shows the position of social telerehabilitation within the general scope of telehealth.

The Latvian national science program VPP INOSOCTEREHI is a new three-year multidisciplinary project in the field of social telerehabilitation focused on social inclusion. This project is being conducted by four Latvian Universities (Rezekne Technology Academy, Latvia University, Riga Technical University, and Liepaja University), and investigates the use of mobile technology in the scope of social rehabilitation (http://telerehabilitation.lv/).

This paper reports on reflections derived from preliminary and explorative desk research conducted within the VPP INOSOCTEREHI scientific program. It focuses on several aspects related to accessibility and usability of telerehabilitation services.

## ACCESSIBILITY TO SOCIAL TELEREHABILITATION SERVICES

The aim of our research, which is part of the VPP INOSOCTEREHI scientific program, is to collect solutions, best practices, and suggestions useful in the design, implementation, and assessment of user interfaces that can maximize usability in social telerehabilitation systems for older adult users.

It is important to underline that social telerehabilitation, which is intended as the production of new solutions to social rehabilitation problems in more effective, efficient, and sustainable ways, is a new field of investigation.

Literature shows that the goals of social telerehabilitation services for older adults essentially are (Vichitvanichphong, Talaei-Khoei, Kerr & Ghapanchi, 2014):

- reducing growth of long-term care needs;
- increasing access to and quality of care services;
- increasing productivity of assistance personnel involved;
- reinforcing the efficiency of long-term care systems.

There is a general consensus that new technologies can help older adults improve their quality of life. Many authors emphasize that technology can allow older adults to maintain their independence and autonomy, assist them to stay healthy, and counteract their reduced capabilities.

However, in most cases patients merely appear as input data for computer-based systems, health-care professionals, and policy-makers. There are comparatively fewer ICT-based systems and applications designed to directly benefit older adults.

Moreover, most computer programs for the care of older adults can help in alleviating the caregivers’ burden, even allowing them to perform tasks from a distance:

- Health monitors which continuously monitor pulse, skin temperature, and movement;
- Video monitors;
- Hip protectors;
- Pressure mats which check if a person mobilises from a bed or a chair;
- Door alerts for patients who wander at night;
- Movement detectors.

## CHALLENGES FOR SOCIAL TELEREHABILITATION SERVICES

The benefits obtained through the use of ICT could be extended to older adults’ social lives. A crucial focus of social telerehabilitation is to regain people’s psychophysical functions and to improve their everyday quality of life. Mostly, it has been argued that ICT can improve the quality of life of lonely and isolated individuals in late adulthood (Vedel, Akhlaghpour, Vaghefi, Bergman & Lapointe, 2013).

In the last few years the number of older adults using new technologies has been increasing, although their technology usage remains notably less than that of the younger generation (Feist, Parker, Howard & Hugo, 2010; Zickuhr & Madden, 2012). Nonetheless, whilst older adults may be an emerging group, they have significant potential as ICT consumers. New services designed both to facilitate older adults’ access to health services and to interact with their family and friends and cultivate relationships are multiplying (Boot et al., 2013).

Despite this, from our research it emerges that some factors hold back the use of technology by older adults. The principal factors are the cost of the technology, followed by its complexity (i.e., in terms of the skills required to be able to use it).

A very real problem in designing effective ICT-based solutions for older adults arises from the fact that they don’t form a homogeneous class. Accordingly, when designing new services, developers need to both pay attention to the characteristics of particular sub-classes of older adult users, and personalize their interactions with these services. This doesn’t facilitate the implementation of telerehabilitation services for older adults as a whole (Steele, Lo, Secombe & Wong, 2009; Wang, Rau & Salvendy, 2011; Lee, 2014).

### DESIGNING SOCIAL TELEREHABILITATION SERVICES FOR OLDER ADULTS

The literature largely assumes that age acts as an impediment or disadvantage when using ICT applications (Pak, Price & Thatcher, 2009; Zimmer, Tams, Craig, Thatcher & Pak, 2015). For example, human memory declines with age, and although experience increases, inflated self-confidence may lead to mistakes. As a consequence, age, lack of domain knowledge, and less usable interfaces may discourage the use of ICT based services. This means that the design of telerehabilitation services for older adults is not simple, and is sometimes very challenging.

**Figure 2:**
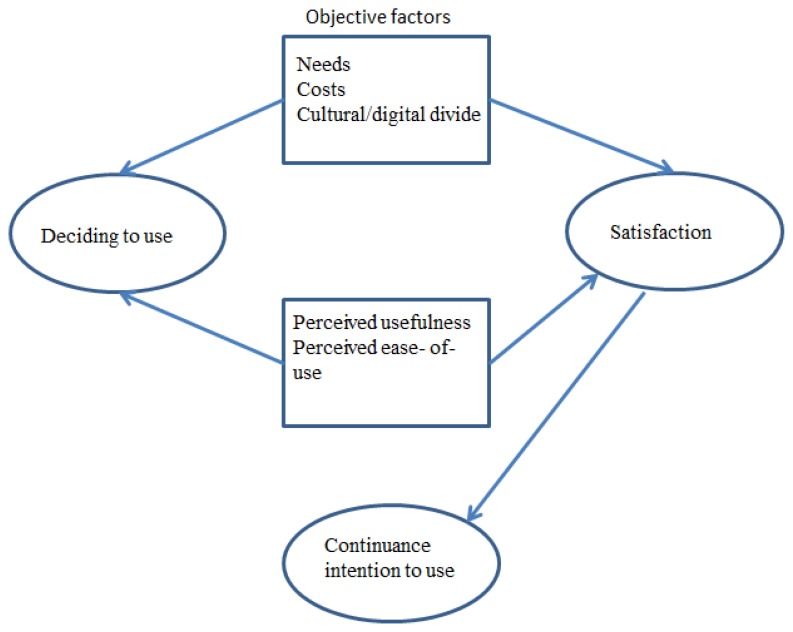
Factors influencing the use of a technology.

We identified some factors that affect the use of ICT solutions by older adults. The principal factor concerns the expected benefits. Particularly if the older adult is low skilled, they have to be persuaded about the real need for using an ICT application. To overcome the cultural resistance to computers, the application should demonstrate that they are both necessary and economically advantageous. Figure 2 outlines the principal factors affecting the decision to use a certain technology, and for continuing to use it.

In general, perceived/supposed usefulness and perceived/supposed usability are two key factors for choosing an ICT application. *Perceived ease-of-use* (or *perceived usefulness*) is a very important concept in the Technology Acceptance Model. It is defined as the individual’s belief that a certain system is free of complexities (Lee, 2010), and is deemed to have a direct impact on user behaviour. It has been noted that often, the individual’s belief as to the ease of use of a technology has an impact on his/her perception of the usefulness of that technology (Vijayasarathy, 2004). For example, perceived usefulness may influence an individual to believe that using a certain ICT application adds value to his/her life (Limayem & Cheung, 2008). For this reason, perceived usefulness is a key factor in the design of telerehabilitation services, since it can influence the individual’s level of satisfaction and, in turn, their intention to make use of these services. *Satisfaction* is the term used to describe a person’s positive feeling of fulfilment derived from the perceived performance of a product or service. A user’s intention to continue to use a certain ICT application is related to their level of satisfaction when using it.

### THE IMPORTANCE OF USER INTERFACE

Usability issues may prevent older adults from easily accessing ICT-based services, and usability barriers may differentially affect them. This suggests that the design of ICT applications should be adapted to the age-related changes in physical (sight, hearing, movement) and cognitive capabilities, since these changes limit access to and interaction with ICT-based services.

However, as we have previously highlighted, older adults are not a homogeneous class. Cultural factors and different physical/cognitive conditions create very remarkable disparities amongst older adults, and these disparities have a profound influence on the use of ICT applications and services.

The user interface represents the first obstacle in using a computer application. At present, while most interfaces through which one accesses a computer program are simple, their design is general purpose, meaning that they are assumed to be equally usable by anyone. Moreover, computer program developers take some basic concepts as given. They assume that everyone understands a menu, a dialog box, a list box, a radio button, etc. Computer applications often don’t explain carefully how to achieve a goal and their messages are often cryptic and designed for skilled users. Providing users with an excess of functions complicates matters. The developers’ idea is that giving users a wide range of functions increases their capability and personal freedom. Unfortunately, by doing so, the opposite result is often achieved, and a computer program is actually perceived as overly complex by many users: in short, the more one allows users to do, the less they are able to choose what they really do want to do.

Finally, there is a specific factor that complicates the design of computer applications for older adults. ICT is constantly and rapidly evolving, and its use varies by age. In other words, the new technology skills of those aged 65 years old today cannot be compared to the skills of those who will be 65 in the next 10 years. Future generations of older adults should be more accustomed to ICT use. The real problem for designers is how to manage the transition phase for the users today and in the next few years.

### THE NECESSITY FOR A THEORETICAL FRAMEWORK

We are persuaded that a theoretical framework is needed to tackle the issues that can affect the use of computer applications by older adults.

Respecting the human personality as a whole (mind, feelings, will, attitude, values, motivation, self-experience, specific features of age, etc.) and recognising the necessity of facilitating the older adult’s access to ICT-based services are crucial. Two theories can offer support: social cognitive theory and social ecological theory.

Social cognitive theory (Bandura, 1977) recognises that there are direct interaction links between behaviour, environment, and personal psychosomatic conditions:

- on one hand, the environment determines human behaviour,
- on the other, human behaviour and activity change the environment.

Social ecological theory (Bronfenbrenner, 1979) assumes that behaviour is determined by many social subsystems: family, community, workplace, beliefs and traditions, economy, physical environment, and networks of social relations. It is assumed that changes in one subsystem will induce changes in others.

These two theories can provide the basic framework for developing a model that encompasses all of the factors related to the use of computer applications by older adults.

Our opinion is that an experiential learning approach would be useful for designing computer applications that are effective for older adults. However, an operative model should include both an online survey and analysis of attitudes, expectations, and level of satisfaction of users, and the reinforcement of their progress. Specific research is required to investigate how positive reinforcement could influence the use of technology by older adults. Nevertheless, at the moment, the principal preliminary issue we have to tackle is how to measure an individual’s physical/emotional factors that affect their use of technology usage.

## CONCLUSION

This paper introduces some reflections on the issue of technology usage among older adults. We have focused on usability, since it may limit older adults’ access to ICT-based services. We have underlined the need for adopting a theoretical framework, and the necessity that this incorporates an online survey to analyse the attitudes, expectations, and level of satisfaction of older adult users, whilst also taking into account that older adult users do not, in fact, form a homogeneous class.

The next phase of our research will be the collection and analysis of data on older adults’ attitudes, skills, and their perceptions of the usefulness of computer applications, in an attempt to identify some generic classes and trace their typical behaviours.

This step is crucial for the definition of a framework that guides the design of a flexible interface capable to adapt itself automatically to the actual user capabilities (Browne, 2013). Our hypothesis is that it is possible to identify some specific characteristics of older adults and, accordingly, customize the user interface, at least for certain aspects (LeRouge, Ma, Sneha & Tolle, 2013). Moreover, we expect that the definition of classes and user profiles can also be useful for studying cases of older adult behaviour (e.g., the correlation between the data of their interaction with a computer and some pathologies, and *vice versa)*. (Lancioni, 2014).

At the moment, our research focuses on the selection of materials to be used in the experiment, such as service portals, entertainment sites, and computer-based health applications. We will also prepare a set of *ad hoc* user interfaces in order to evaluate their effectiveness on our sample.

In the future, our ambition is to repeat the experiment in different countries and/or for different cultural groups and use the results for modelling virtual assistants (Morandell, Hochgatterer, Fagel & Wassertheurer, 2008; Yaghoubzadeh, Buschmeier & Kopp, 2015) that can help to run more effective rehabilitation services for older adults.

Indeed, our investigation is in the scope of a new generation of healthcare services that should be highly adaptable and customizable to individual contexts facilitating interaction, especially by people with disabilities.

## Figures and Tables

**Figure 1:**
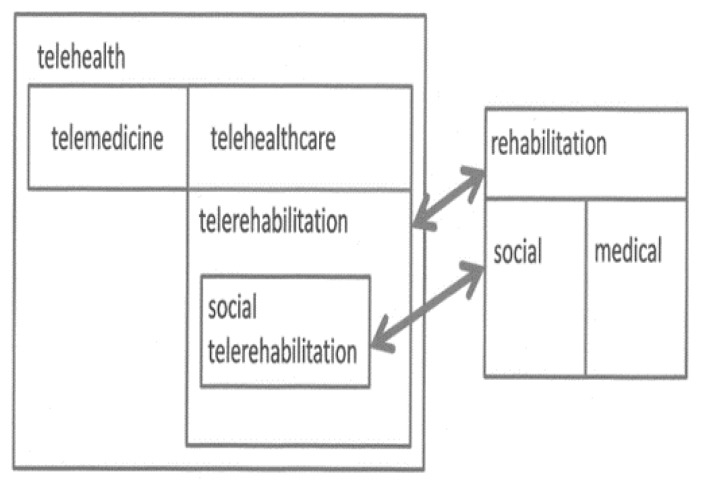
Social telerehabilitation within the telehealth general framework.

## Footnotes

1 The use of social telerehabilitation as an ‘umbrella term’ is intended to be inclusive of the rehabilitative roles that many professions (e.g., psychology, psychiatry, social work, occupational therapy, speech-language pathology, etc.) routinely play in enhancing social communication as part of their professional scopes of practice.
